# Relation- and Task-Oriented Roles as Antecedents of Ethical Leadership: Examining Synergistic Effects

**DOI:** 10.5964/ejop.11891

**Published:** 2024-11-29

**Authors:** H. M. Saidur Rahaman

**Affiliations:** 1 Department of Psychology, Jagannath University, Dhaka, Bangladesh; Dublin City University, Dublin, Ireland

**Keywords:** ethical leadership, opposing domain theory, relationship-oriented role, task-oriented role

## Abstract

A growing body of literature demonstrates that ethical leadership has positive effects on employees’ work outcomes. Ethical leadership upholds the importance of “normatively appropriate conduct through personal actions and interpersonal relationships” (Brown et al., 2005, p. 120; doi:10.1016/j.obhdp.2005.03.002). However, extant empirical research does not answer the question- of how ethical leaders balance their relation maintenance (i.e., relationship-oriented role) and performance maintenance (i.e., task-oriented role) behaviors with their employees to be perceived as ethical leaders. In the present paper, drawing upon the propositions informed by opposing domains theory and related research, I theorize that leaders’ relationship-oriented and task-oriented roles create synergistic effects that predict their employees’ perceptions of ethical leadership. Results across two studies (an experiment and a correlational study involving samples from two different cultures) convergently confirmed the hypothesized relationships. I conclude by discussing several key theoretical and practical implications of these findings.

More than a decade ago, and even in recent years, we witnessed large-scale ethical meltdowns of leaders in global corporations (e.g., Enron, Worldcom, Tyco, Volkswagen) where such leaders compromise ethical issues either for profit or for personal gain. Keeping such examples in mind, leadership scholars have shown tremendous interest in doing research on ethical leadership. Ethical leadership is “the demonstration of normatively appropriate conduct through personal actions and interpersonal relationships, and the promotion of such conduct to followers [employees] through two-way communication, reinforcement, and decision making” (Brown et al., 2005, p. 120). A growing body of research has shown a range of important consequences of ethical leadership for the employee, such as encouraging ethical behavior (Mayer et al., 2012), stimulating employee in-role and extra-role performance (Piccolo et al., 2010), reducing workplace conflict (Mayer et al., 2012), and minimizing employee’s dysfunctional resistance in the time of organizational change (Rahaman et al., 2020).

However, compared to the consequences, research examining the antecedents (i.e., personal antecedent) of ethical leadership has been slowed, which has triggered heightened interest among scholars along this line of research in recent years (Marquardt et al., 2021). Until now, apart from the dispositional trait approach, a relatively small growing body of research has focused on the antecedents of ethical leadership grounded on any specific theoretical perspective to broaden the scholarly understanding of its emergence. A few exceptions are the work of Rahaman et al. (2019), Keck et al. (2020), and Brown & Treviño (2014). Indeed, in a recent meta-analytic review, Ko et al. (2018) have urged doing further research examining the antecedents of ethical leadership, such as different leader characteristics, so that knowledge can be garnered to develop and cultivate such leadership in the organization. One such characteristic can be the leadership role (i.e., relationship-oriented and task-oriented roles, Bales, 1958), which is distinct from leadership style but can be enacted by the same (see Boyatzis et al., 2014).

Any task a leader may want to accomplish can have a relational component (i.e., relationship-oriented role) and an analytical component (i.e., task-oriented role), which can be done in ethical/unethical ways (Boyatzis et al., 2014; Rochford et al., 2017; Yukl, 2012). A leader’s sole focus on the relationship-oriented role can hamper his/her concentration toward meeting the clearly defined goals at work, such as lapses in attention and performance errors, which can reduce the performance on the analytic task (Boyatzis et al., 2014; Fassbender et al., 2009; Mason et al., 2007; McKiernan et al., 2003). However, the application of conscious reasoning, as demonstrated in attention toward analytical tasks, is essential for any leader to be an ethical leader (Greene, 2007; Jordan et al., 2013). Similarly, a leader’s sole focus on the task-oriented role may make the leader prone to be a Machiavellian thinker, which can have negative implications for ethical leadership (Bagozzi et al., 2013; Brown & Treviño, 2006) as in such instances he/she may fall short for openness to new ideas, people, emotions, and ethical concerns, which are important to any organization’s success (Boyatzis et al., 2014; Marquardt et al., 2021). Specific to ethical leadership, people orientation and role clarification are two important behavioral components of ethical leadership (Kalshoven et al., 2011), which are argued to be consecutively similar to relationship-oriented and task-oriented behaviors (Yukl et al., 2013). People orientation mirrors “genuinely caring about, respecting, and supporting subordinates and where possible ensuring that their needs are met” whereas role clarification mirrors “transparency in clarifying performance goals and expectations” (Kalshoven et al., 2011, p. 53). Hence, it is likely that ethical leaders need to trade off between a relationship-oriented role and a task-oriented role.

While ethical leadership stresses normatively appropriate conduct through personal actions and interpersonal relationship (Brown et al., 2005), any empirical research has not yet addressed whether ethical leaders focus more on the satisfaction, motivation, and general well-being of the employees (i.e., relationship-oriented role, Bales, 1958) or they focus more on the tasks that need to be carried in order to meet certain goals, or to achieve certain performance standards (i.e., task-oriented role, Bales, 1958)? In other words, how do they balance between these two leadership roles to be perceived as an ethical leader? The answer to this question is vital as it can provide a thorough understanding of the relational predictors of ethical leadership, which have practical implications for training and developing such leadership behavior in the organization (Waldman et al., 2017). Moreover, unraveling the mechanism of how leaders can increase their ethical leadership perceptions of their employees can be vital as such leadership is crucial for organizations’ long-term success and cost-saving (see Marquardt et al., 2021).

In the present study, I ground on the propositions informed by a neuroscientific theory, namely opposing domain theory (Jack et al., 2013) and related theoretical pieces (Boyatzis et al., 2014; Rochford et al., 2017) to seek an answer to the question as mentioned above. Opposing domain theory describes the antagonistic nature of two modes of human cognition consisting of two different neural networks—the default mood network (DMN), which constitutes the socioemotional reasoning of the brain, and the task-positive network (TPN), which constitutes analytic reasoning of the brain. The theory further suggests that a moral dilemma creates a fundamental tension between these two neural networks (Jack et al., 2014). To accomplish any given task, the activity of one network inhibits the other (Jack et al., 2013). That is, the involvement in analytic tasks would impede our ability to engage in social or relational reasoning and vice versa. However, their negative correlation only means that the activity of one’s inhibits the other. And leaders can be highly analytical and attentive to their followers’ relationship needs. Following the theory and building upon the insights of classic Ohio State Leadership Studies, scholars have argued that these two networks, at the psychological level, consecutively correspond to relationship-oriented and task-oriented leadership roles, and switching between these two roles is also possible (see Boyatzis et al., 2014), and they can be positively related (Breevaart & de Vries, 2021). Furthermore, scholars have also argued that leaders need to make a dynamic balance by switching between these two neural networks, which correspond to their respective leadership roles, to emerge as ethical leaders (Rochford et al., 2017).

Given the above, in the present paper, across two studies, I theorize that there are synergistic effects between relationship-oriented and task-oriented roles, which can predict employees’ perception of ethical leadership. Putting it differently, both the relationship-oriented role and the task-oriented role influence each other in predicting ethical leadership. This influence on ethical leadership is optimized when both relationship-oriented and task-oriented roles are at a high level.

The present study offers a number of noteworthy contributions to ethical leadership research. First, I address the call of Ko et al. (2018) by examining leader characteristics as the antecedents of ethical leadership through the lens of relationship maintenance behaviors of the leaders (i.e., relationship-oriented and task-oriented roles) with their employees to effectively cultivate ethical leadership at the workplace. As such, gaining further insight into how ethical leadership can be trained and developed in the organization has been urged in recent research (Marquardt et al., 2021). Second, I elaborate on and make an empirical contribution by theorizing and examining the synergistic effects between relationship-oriented and task-oriented roles in predicting ethical leadership, which have been assumed in theoretical pieces (i.e., Boyatzis et al., 2014; Rochford et al., 2017). In doing so, I meaningfully extend the line of research that suggests mainly focusing on cognitive/analytic reasoning capacity building for ethical leadership training and development purposes while apparently overlooking the necessity of socioemotional reasoning (i.e., relationship-oriented role) in this regard (Brown & Treviño, 2006; Jordan et al., 2013; Wells & Schminke, 2001). Third, I empirically test and expand some propositions that have been theorized based on the findings of a neurological theory (Boyatzis et al., 2014; Rochford et al., 2017) at the psychological level. Such an approach provides an integrative perspective and complementary level of analysis (Marshall, 2009). Along this line, de Jong (2002) further notes, “the change of meaning of concepts and theories in psychology, and the interpretation of anatomy and biochemistry in psychological terms is a major asset of interlevel synchronous investigation. It allows both top-down and bottom-up influences on theorizing” (p. 457). Consequently, my approach enhances the robustness and explanatory power concerning the theory development process to expand the current understanding of the antecedents of ethical leadership (Butler et al., 2017; Rochford et al., 2017).

## Theoretical Review and Hypothesis Development

### Implications of Opposing Domain Theory for Ethical Leadership Theorization

Applying opposing domain theory at the psychological level, leaders may feel the tension to be an analytical (focusing on the task-oriented role that underlies DMN) or a socioemotional (focusing on the relationship-oriented role which underlies on TPN) leader, which has ethical implications (i.e., ethical leadership) at work as any task a leader needs to accomplish has both relational component and analytical component which can be done in ethical/unethical ways (Boyatzis et al., 2014; Rochford et al., 2017; Yukl, 2012). While there can be tension in focusing on task-oriented or relationship-oriented roles to be an ethical leader, switching between these two roles is possible, and they can be positively related at the psychological level (Boyatzis et al., 2014; Breevaart & de Vries, 2021; Rochford et al., 2017). However, focusing solely on the relationship-oriented role—making sense of leaders’ own and other people’s emotions around them to help in constructing a sense of purpose or vision for the group through their assistance—may hamper concentration on meeting the clearly defined goals at organizations such as lapses in attention and performance errors which reduces the performance on the analytic task (Boyatzis et al., 2014; Fassbender et al., 2009; Mason et al., 2007; McKiernan et al., 2003). This reduced attention toward analytical tasks may have negative implications for ethical leadership as it requires the application of conscious reasoning to reach a justifiable moral verdict (Greene, 2007; Jordan et al., 2013). Likewise, focusing solely on emphasizing task-oriented role—financial planning, metrics, forecasting, problem solving, and strategic engagement intending to accomplish task achievement—may lead to harmful effects when openness to new ideas, people, emotions, and ethical concerns are important to an organization success (Boyatzis et al., 2014). Particularly, it may make the leader prone to be a Machiavellian thinker, which has negative implications for ethical leadership (Bagozzi et al., 2013; Brown & Treviño, 2006). Instead of prioritizing the long-term and big picture importance of fulfilling fairness and justice, these leaders tend to put importance on short-term strategic advantage. For example, a decision that seems feasible in terms of budget analysis may not be the best decision for the company in the long run (Boyatzis & Jack, 2018). Recent research has demonstrated the direct role of DMN in predicting ethical leadership (Waldman et al., 2017). Schaich Borg et al. (2011) further show that the weighting of moral consideration is associated with the regions of DMN, while making the moral verdict is associated with the regions of TPN. At the psychological level, this finding indicates the requirement of both socioemotional (i.e., relationship-oriented role) and analytical (i.e., task-oriented role) reasonings to make an ethical decision. Hence, translating the above mentioned arguments in the case of ethical leadership, I expect that relationship-oriented and task-oriented roles positively predict ethical leadership. Therefore, I hypothesize:

**H1a.** *Leaders’ relationship-oriented role will positively predict ethical leadership.*
**H1b.** *Leaders’ task-oriented role will positively predict ethical leadership.*

Neuroscience research shows not only parallel but also the interactive nature of the dual processing (reason and emotion) in our brain (Satpute & Lieberman, 2006). Furthermore, a synergistic interaction may occur between emotion (i.e., relationship-oriented role) and reason (i.e., task-oriented role) as they are interdependent (see De Caro & Marraffa, 2016). Indeed, at the psychological level, Weed et al. (1976) have shown evidence of interaction between task type and employee preferences for task vs. relationship-oriented leaders (as cited in Boyatzis et al., 2014). The tri-dimensional leadership model of Hersey and Blanchard (1969) also outlines the outcomes of different combinations of relationship and task behaviors. Research in consumer behavior and social behavior also shows the interaction between cognition (i.e., analytic reasoning) and emotion (Gutnik et al., 2006; Shiv & Fedorikhin, 1999). Specific to ethical leadership, scholars have grounded on the insights of opposing domains theory and gone beyond to theorize at the psychological level that it is necessary to have a dynamic balance between relationship-oriented and task-oriented roles for a leader to emerge as an ethical leader as such, privileging one perspective over another may result in ethical failure while both of them are required to be an ethical leader (Boyatzis et al., 2014; Rochford et al., 2017). Such dynamic balance implies the presence of an interaction between relationship-oriented and task-oriented roles. Specifically, the pattern of such interaction can be synergistic as either role alone cannot optimize the emergence of ethical leadership (c.f., Rousseau et al., 2008). Hence, I further theorize the presence of synergistic interactions between relationship-oriented and task-oriented roles in predicting ethical leadership. Cohen et al. (2003) define synergistic interaction as when “both predictors affect the [outcome] in the same direction, and together they produce a stronger than additive effect on the outcome” (p. 285). That is, the effect of a relationship-oriented role can be influenced by the presence of a task-oriented role and vice versa. Hence, they can reinforce (i.e., amplification of positive effect) each other in predicting ethical leadership (c.f., Gerpott et al., 2020). Taken together, I formulate the following hypotheses:

**H2.** *There is an interaction between relationship-oriented and task-oriented roles, which predict ethical leadership.*
**H3a.** *Task-oriented role moderates the relationship between relationship-oriented role and ethical leadership, such that the relationship is strongest when the task-oriented role is high (vs. low).*
**H3b.** *Relationship-oriented role moderates the relationship between task-oriented role and ethical leadership, such that the relationship is strongest when the relationship-oriented role is high (vs. low).*

### Overview of Studies

In the present research, I test my proposed hypotheses across two studies employing different designs and samples. Initially, I conducted a vignette study involving participants to explore the causal nature of the stated Hypotheses 1 and 2 (see Aguinis & Bradley, 2014). As such, I manipulated the relationship-oriented role and the task-oriented role and asked the participants to rate their perception of ethical leadership. In the second study, I conducted a cross-sectional survey with employees to replicate the findings of the first study (Hypothesis 1 and Hypothesis 2) further and test Hypothesis 3. In designing the second study, I particularly rely on the research that has shown that leader-observer ratings are of similar magnitudes for relation- and task-oriented behaviors (Lee & Carpenter, 2018) and took the followers’ rating of their perceptions of their leaders’ relation- and task-oriented roles. Indeed, followers’ perceptions of the leader are indispensable for the construction of leadership (see Oc et al., 2023). Altogether, the two studies demonstrate the evidence of internal and external validity for the tested hypotheses.

## Study 1

### Method

#### Participants

One hundred and ten students from a large European university were invited to participate in this study voluntarily. The author individually approached each participant in one of the university libraries and a student social club. One hundred one participants responded, which constitutes a response rate of 91.82%. The respondents consist of 43 females and 58 males with an average age of 22.27 (*SD* = 2.85) years. 47 of them were bachelor’s students, while 54 were master’s students. 63 had work experience either as part of an internship, a part-time job, or weekend or holiday jobs. Work experience averaged 10.91 (*SD* = 17.59) months.

#### Procedure and Measures

Based on previous scenario experiments, which provide a clear behavioral description of the task-oriented and relationship-oriented leadership roles (Cohen et al., 2004; Ehrhart & Klein, 2001), I manipulated the two leadership roles resulting in a 2 x 2 i.e., (relationship-oriented role: high vs. low) x (task-oriented-role: high vs. low) between-subjects factorial design. Participants were randomly allocated to the conditions. At the beginning of the vignette, participants were asked to answer demographic questions about their age, sex, education, and work experience. Then, they were instructed to read the text, which describes the behavior of a leader named David working in the fictitious company Abro, which consists of one of the four conditions of the vignette as per the factorial design. The description of all four conditions is given in Table 1.

**Table 1:** Description of the Four Conditions of the Vignette Experiment

High Relationship-Oriented Condition	High Task-Oriented Condition
*The first thing David emphasizes in his interaction with employees is to treat them with kindness and consideration. He is committed to being friendly and respectful, even when stress is high or when there is a lot of work. Another thing he emphasizes is to keep employees informed of progress on projects or any other organizational issues that might affect them, and he is always available to listen to employees’ problems, whether these are personal or work-related. In addition, he shows trust and confidence in his employees. He wants them to feel involved in their work and to know that he thinks they can do a good job. The final thing he does with his employees is that he recognizes their contributions. If they work hard and do a good job, he goes out of his way to make sure they know that their work is appreciated.*	*David begins by working with the employees to set goals for their work. He does not want to overwhelm them with unachievable standards, so he makes sure their goals are realistic yet still challenging. He is very careful and detailed in laying out what employees need to get done. Once they know what specific objectives are, he makes sure that employees have the necessary tools to successfully meet their goals. He provides them with the necessary supplies, equipment, and technical assistance to ensure that they can be successful. Finally, he coordinates the work so that employees and their fellow subordinates know what their job content is and there is no overlap between the two. David wants everyone to know what his or her role is so that he or she can see how he or she is contributing to the accomplishment of the organization’s goals.*
Low Relationship-Oriented Condition	Low Task-Oriented Condition
*David does not emphasize maintaining all his interactions with his employees in a way to treat them with kindness and consideration. He is not committed to being friendly and respectful when stress is high or when there is a lot of work to be done. Another thing he does not emphasize is to keep employees informed of progress on projects or any other organizational issues that might affect them, and he is mostly not available to listen to his employees’ problems, whether these are personal or work-related. In addition, he does not show trust and confidence in his employees. He does not want them to feel involved in their work and to know that he thinks they can do a good job. The final thing he does with his employees is that he does not care about recognizing their contributions. If they work hard and do a good job, he does not go out of his way to make sure they know that their work is appreciated.*	*David does not begin by working with the employees to set goals for their work. He wants to overwhelm them with unachievable standards, which make it seem to them that their goals are unrealistic and very challenging. He is very careless and not detailed in laying out what employees need to get done. Once they know what specific objectives are, he does not make sure that employees have the necessary tools to successfully meet their goals. He does not provide them with the necessary supplies, equipment, and technical assistance to ensure that they can be successful. Finally, he does not coordinate the work so that the employees and their fellow subordinates know what their job content is, and there is no overlap between the two. David does not care whether everyone knows what his or her role is so that he or she can see how he or she is contributing to the accomplishment of the organization’s goals*

After reading the scenario, participants were instructed as follows: “Based on the above descriptions of the leader, David, please answer the two questions below: To what extent do you think David puts importance on managing the relationship with employees at work? And to what extent do you think David puts importance on assisting in the tasks employees have in their work?” Consecutively, these two questions were used as manipulation checks for relationship-oriented and task-oriented leadership roles. The manipulation check items were grounded on a 7-point Likert-type scale ranging from 1 (*Not at all important*) to 7 (*Extremely important*). After rating the manipulation questions, participants were instructed as follows: “Now imagine that you are working with David, and he is your boss. How will you rate him as your supervisor?” Participants rated the 10-item ethical leadership scale of Brown et al. (2005). I used a 5-point Likert-type scale ranging from 1 (*Strongly disagree*) to 5 (*Strongly agree*). Sample scale items included: ‘David conducts his personal life in an ethical manner’ and ‘makes fair and ethical decisions.’ In the present study, the Cronbach alpha reliability of the scale was .89.

### Results

#### Manipulation Check

I ran a 2 x 2 ANOVA on the relationship-oriented role manipulation check, which revealed a significant main effect for a relationship-oriented role manipulation on relationship-oriented role manipulation check, *F*(1, 97) = 402.66, *p* < .001, partial η*^2^* = .81. This indicated that participants perceived that their leader is having a high relationship-oriented role in the high relationship-oriented condition than in the low-relationship-oriented condition (see Table 2). Unexpectedly, task-oriented role manipulation also showed a significant main effect on the relationship-oriented role manipulation check, (1, 97) = 5.02, *p* < .05, partial η*^2^* = .05. However, the degree of spillover was rather small compared with the magnitude of the intended effect. Specifically, the *F* statistic for the effect of relationship-oriented role manipulation on the relationship-oriented role manipulation check was almost 80 times larger than the statistic for task-oriented role manipulation on the relationship-oriented role manipulation check, and the partial η*^2^* value was over 16 times larger. Hence, relationship-oriented role manipulation appeared to be successful and unlikely to impair the interpretations of the main results of this experiment (see He et al., 2017). The interaction effect was not significant, *F*(1, 97) = .11, *p* = .74, partial η*^2^* = .001.

**Table 2:** Mean Predicted Ethical Leadership as a Function of Relationship-Oriented and Task-Oriented Roles in Study 1

	Low relationship-oriented role	High relationship-oriented role
Low task-oriented role	1.95(.46)	2.81(.60)
High task-oriented role	2.38(.49)	3.80(.41)

*Note. N* = 101. Higher ratings indicated higher ethical leadership. Standard deviations are provided within parentheses.

A 2 x 2 ANOVA on the task-oriented role manipulation check revealed a main effect of task-oriented role manipulation, *F*(1, 97) = 165.10, *p* < .001, partial η*^2^* = .63. Participants in the high task-oriented role condition rated the leader higher on task focus than participants in the low task-oriented condition (see Table 2). Unexpectedly, the main effect of relationship-oriented role manipulation was also significant for task-oriented role manipulation check, *F*(1, 97) = 7.41, *p* < .01, partial η*^2^* = .07. However, the degree of spillover was rather small compared with the magnitude of the intended effect. In particular, the *F* statistic for the effect of task-oriented role manipulation on the task-oriented role manipulation check was almost 22 times larger than the statistic for relationship-oriented role manipulation on the task-oriented role manipulation check, and the partial η*^2^* value was over 9 times larger. Hence, task-oriented role manipulation appeared to be successful and unlikely to impair the interpretations of the main results of this experiment (see He et al., 2017). The interaction effect was not significant, *F*(1, 97) = .19, *p* = .67, partial η*^2^* = .002.

#### Hypothesis Testing

**Figure 1:**
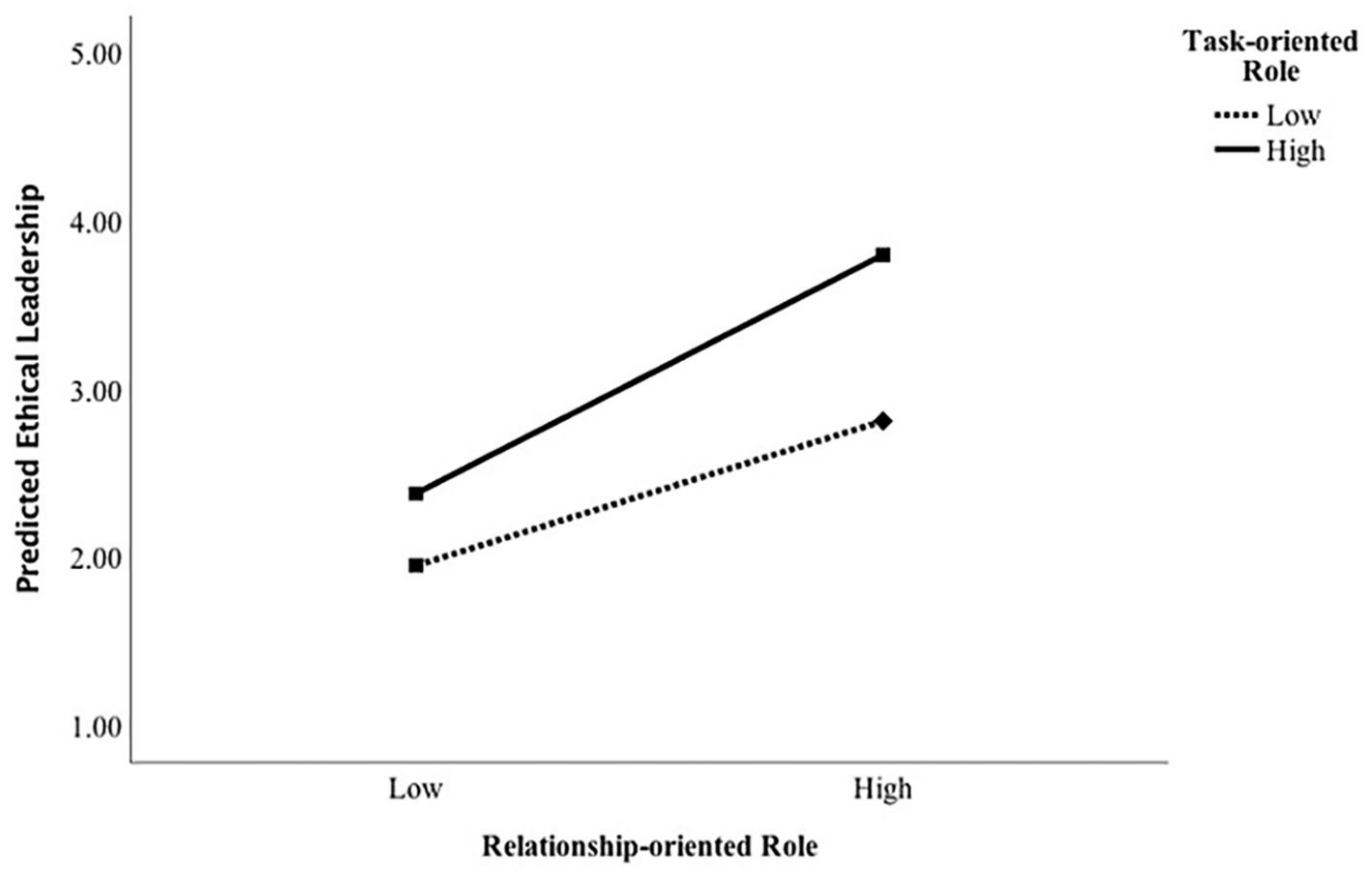
Interaction Between Relationship-Oriented and Task-Oriented Roles on Participants’ Perceptions of Ethical Leadership in Study 1

A 2 x 2 ANOVA revealed a significant main effect for relationship-oriented role on ethical leadership, *F*(1, 97) = 134.05, *p* < .001, partial η*^2^* = .58. I also found a significant main effect of task-oriented role on ethical leadership, *F*(1, 97) = 51.76, *p* < .001, partial η*^2^* = .35. This revealed that in the high relationship-oriented-role condition, participants perceived the leader as higher on ethical leadership as compared to the low relationship-oriented role condition. In the high task-oriented role condition, participants perceived the leader as higher on ethical leadership compared to the low task-oriented condition (see Table 2). Thus, the results confirmed my proposed Hypothesis 1a and Hypothesis 1b. A significant interaction effect was also found between relationship-oriented and task-oriented conditions on ethical leadership, *F*(1, 97) = 8.10, *p* = .005, partial η*^2^* = .08 (see Figure 1). These results confirmed Hypothesis 2.

## Study 2

### Method

#### Participants and Procedure

I aimed at getting a heterogeneous sample to be able to tap employees’ perceptions of leadership roles from a wide range of organizations. I followed past research to attain this aim and recruited participants by using the snowball sampling technique (see Keck et al., 2020, for a similar approach). I invited the participants via personal messages on social network sites to voluntarily participate in a web-based survey. I further requested the participants to invite further participants via their personal network. I took several steps to make sure that the surveys were completed accurately. At the beginning of the survey, I provided complete instructions about the nature and scope of the study in the first place and then what the participants needed to do to fill out the survey form accurately (i.e., not skipping any survey items). I ensured the participants the confidentiality and anonymity of their responses.

I got responses from 131 participants from a wide range of organizations situated in a South Asian country (e.g., Banking, Insurance, Education, Financial institutions, etc.). In my sample, the participants averaged 29.73 years of age (*SD* = 4.45) and 3.64 years (*SD* = 3.07) of work experience. 71.80% of them were male. Of them, 0.80% had an MPhil/PhD degree, 3.80% had a master’s degree, 25.90% had a bachelor’s degree, and 69.50% had a higher secondary education certificate.

#### Measures

I followed the standard back-translation procedures to translate all the study measures from English to Bangla (Brislin, 1986). Unless otherwise stated, all the measures were grounded on a five-point Likert-type scaling (1 = *Strongly disagree* to 5 = *Strongly agree*).

##### Ethical Leadership

Participants rated a ten-item ethical leadership scale developed by Brown et al. (2005). A sample item was “My supervisor has the best interest of employees in mind” (1 = *Strongly disagree* to 5 = *Strongly agree*).

##### Relationship-Oriented Role

To assess leaders’ relationship-oriented role, participants rated four items used in a previous study (Ehrhart & Klein, 2001). The items are: My supervisor treats subordinates with kindness and respect, emphasizes communication with and listening to subordinates, shows trust and confidence in subordinates, and provides recognition and shows appreciation for subordinates’ contributions and accomplishments.

##### Task-Oriented Role

Participants rated four items used in Ehrhart and Klein’s (2001) study. The items are: My supervisor- guides subordinates in setting performance goals that are high but realistic, plans and schedules at work, provides necessary supplies, equipment, and technical assistance, and coordinates subordinate activities.

#### Control Variables

Research showed that people’s gender, age, and education are related to their (un)ethical intention and behavior (Kish-Gephart et al., 2010), which may confound the interpretations of my findings for ethical leadership (Bernerth & Aguinis, 2016). Consequently, I first checked the correlations of these variables with employees’ perceptions of ethical leadership (i.e., dependent variable) and found no significant correlations between these variables and ethical leadership. Hence, as per the recommendations of Becker (2005) and Carlson and Wu (2012), I did not control these variables (i.e., impotent control variable) in the case of hypothesis testing. That is, I ran the analysis excluding the control variables.

#### Confirmatory Factor Analysis

I conducted a series of confirmatory factor analyses to examine the distinctiveness and validity of the study measures. To do so, I included all the items of the study measures. Also, while running the model, I neither correlated any items pertaining to their corresponding specific factors nor the error terms. A three-factor model (χ*^2^* = 224.98, *df* = 132, *p* < .001, CFI = .90, RMSEA = .07, SRMR = .06) demonstrated a good fit to the data (Hu & Bentler, 1999). A two-factor model revealed a relatively poor fit to data (χ*^2^* = 238.25, *df* = 134, *p* < .001, CFI = .89, RMSEA = .08, SRMR = .06). Furthermore, a one-factor model (χ*^2^* = 278.66, *df* = 135, *p* < .001, CFI = .85, RMSEA = .09, SRMR = .07) revealed relatively worse fit to data. Overall, these indices support the distinctiveness of the three measured variables rated by the employees.

#### Common Method Variance Test

Though research showed that the interactive effect (as proposed in Hypothesis 2) could not be the artifact of common method variance (Siemsen et al., 2010), I performed two tests to examine the issue further. First, I did a Harman single-factor test. The test results showed that a single factor could explain 40.67% variance, which is less than 50%. Hence, common method variance (CMV) does not seem likely to be a problem in this data set (Podsakoff & Organ, 1986). Second, I followed a CLF (Common Latent Factor) approach to test for common method bias. I compared the value of standardized regression weights with and without having the CLF in the three-factor CFA model. I found that the differences in the regression coefficient values for having and not having CLF in the CFA models were as follows: values of 15 items were less than 0.10, the value of 1 item was 0.10, and the values of the two items were less than 0.15. All of these value differences are less than the threshold value of 0.20 to raise concerns for common method bias (StatWiki, 2022). These results showed that CLF is not taking away a substantial amount of variance from the observed items in the CFA model, which indicated that CMB is less likely to be an issue for this dataset (see Podsakoff et al., 2003).

### Results

The descriptive statistics, correlations, and Cronbach alphas of the measured variables are presented in Table 3. I used OLS regression analyses to test the hypotheses. Consistent with Hypothesis 1, results revealed a significant relation between relationship-oriented role and ethical leadership, β = .54, *t*(127) = 7.62, *p* < .001 (supported Hypothesis 1a) and a significant main effect for the task-oriented role on ethical leadership, β = .31, *t*(127) = 4.28, *p* < .001 (supported Hypothesis 1b) (see Table 4). I computed an interaction term by multiplying the mean-centered scores of the relationship-oriented role and task-oriented role and also regressed it on ethical leadership. An interaction effect between relationship and task-oriented roles was also found β = .13, *t*(127) = 2.07, *p* < .05, which supported the proposed Hypothesis 2 (see Table 4). Furthermore, the inclusion of the interaction term in the regression models significantly increased the explained variance in ethical leadership by 2% (see Table 4).

**Table 3:** Descriptive Statistics, Zero-Order Correlations, and Reliabilities of Study 2

Variable	*M*	*SD*	1	2	3
1. Relationship-oriented role	3.31	1.05	.79		
2. Task-oriented role	3.44	.84	.50*	.61	
3. Ethical Leadership	3.14	.88	.67*	.55*	.89

*Note. N* = 131. Alpha coefficients are noted in the diagonal. The Cronbach Alpha value for the task-oriented role .61 can be regarded as acceptable considering the composite nature of the scale (cf., Hair et al., 2010; Peterson, 1994).

**Table 4:** Results of Regressing Relationship-Oriented and Task-Oriented Roles on Ethical Leadership in Study 2

Predictor	*B*	*SE*	β
Step 1			
Relationship-oriented role (RO)	.60	.05	.67**
Task-oriented role (TO)			
RO x TO			
*Adjusted R^2^*		.44**	
*ΔR^2^*			
*F change*			
*df*		129	
Step 2			
Relationship-oriented role (RO)	.44	.06	.53**
Task-oriented role (TO)	.29	.07	.28**
RO x TO			
*Adjusted R^2^*		.50**	
*ΔR^2^*		.06**	
*F change*		15.52**	
*df*		128	
Step 3			
Relationship-oriented role (RO)	.45	.06	.54**
Task-oriented role (TO)	.32	.07	.31**
RO x TO	.12	.06	.13*
*Adjusted R^2^*		.51*	
*ΔR^2^*		.02*	
*F change*		4.26*	
*df*		127	

*Note. N* = 131. The table presents the unstandardized b-coefficients and standard errors for centered variables, following the recommendation of Cohen et al. (2003).

**p < .001. *p < .05.

To test Hypothesis 3a and Hypothesis 3b, I further conducted a simple slope difference test (see Figure 2a and Figure 2b) by regressing the interaction term on ethical leadership along with the relationship-oriented role and task-oriented role using the path analytic test in Mplus. A simple slope difference test showed that the positive effect of relationship-oriented role on ethical leadership was significantly higher at a high level (+1SD; *simple slope* = .55, *p* < .001) as compared with the low level of task-oriented role (-1SD; *simple slope* = .35, *p* < .001, *simple slope difference* = .19, *p* < .05), supporting Hypothesis 3a.

**Figure 2a:**
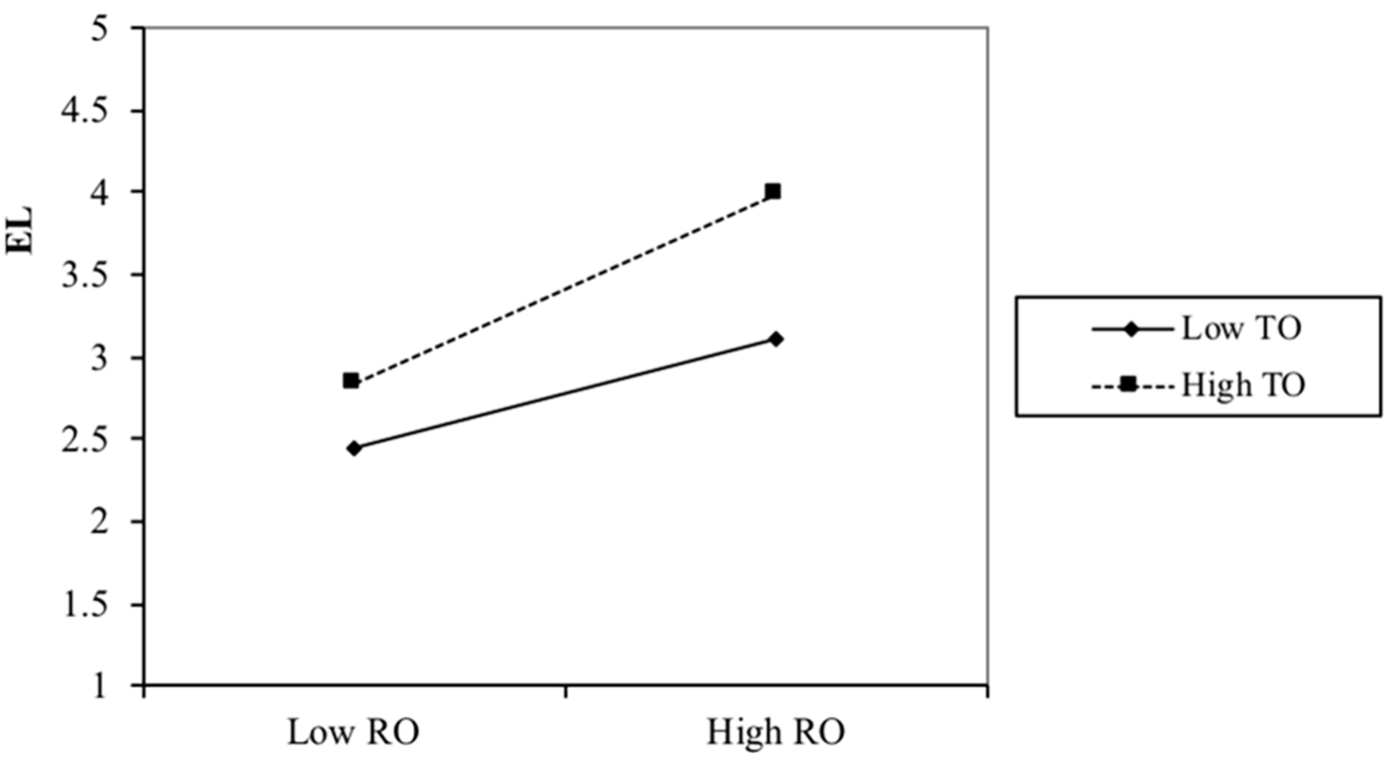
The Moderating Effect of Task-Oriented Role on the Relationship Between Relationship-Oriented Role and Ethical Leadership in Study 2 *Note.* EL Ethical Leadership, RO = Relationship-Oriented Role, TO = Task-Oriented Role.

**Figure 2b:**
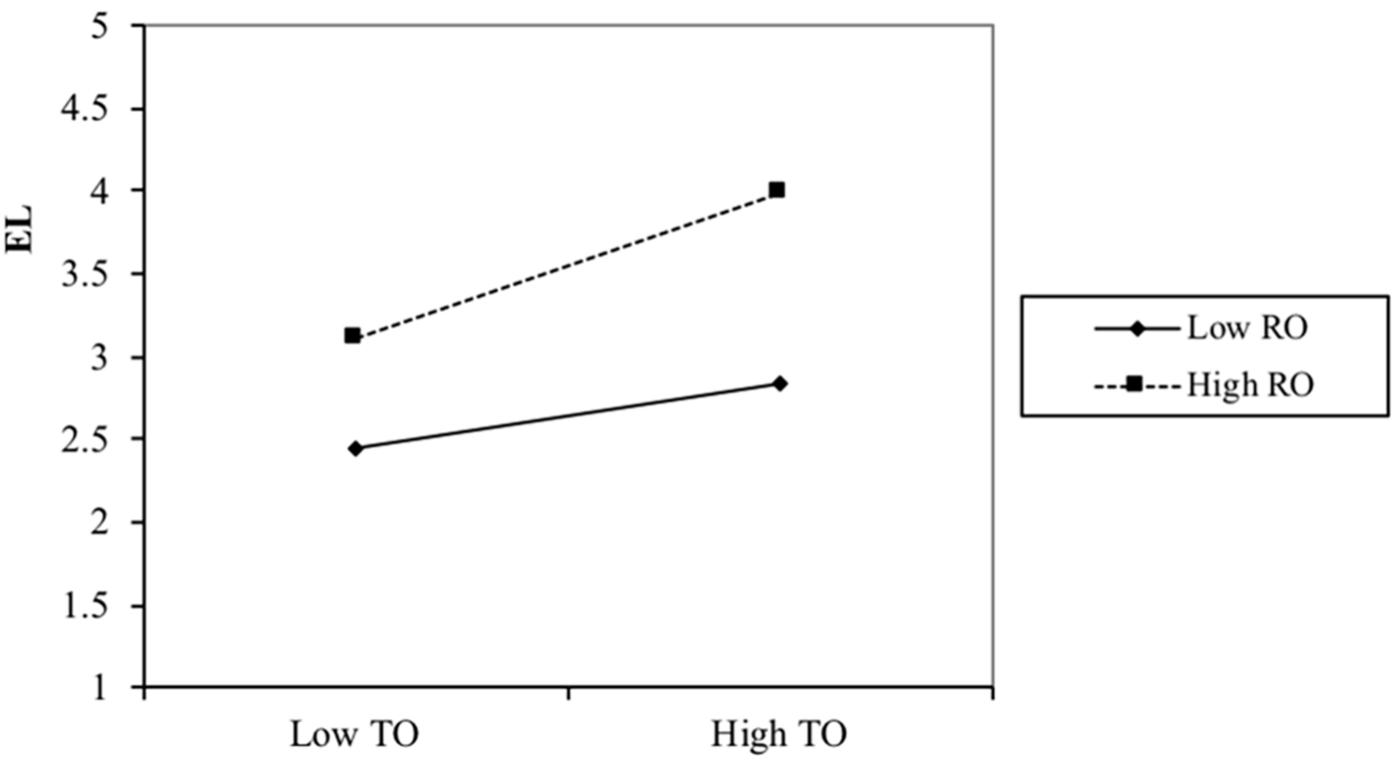
The Moderating Effect of Relationship-Oriented Role on the Relationship Between Task-Oriented Role and Ethical Leadership in Study 2 *Note.* EL Ethical Leadership, RO = Relationship-Oriented Role, TO = Task-Oriented Role.

Furthermore, a simple slope difference test showed that the positive effect of the task-oriented role on ethical leadership was significantly higher at a high level (+1SD; *simple slope* = .44, *p* < .001) as compared with the low level of the relationship-oriented role (-1SD; *simple slope* = .20, *p* < .05, *simple slope difference* = .25, *p* < .05), supporting Hypothesis 3b.

## General Discussion

In this paper, I draw on the propositions informed by a neuroscientific theory named opposing domain theory and related research to examine how the synergy between leaders’ relationship-oriented role and task-oriented role operate as the antecedents of ethical leadership. As hypothesized, across two studies, I found support in favor of my theorizing that leaders’ relationship-oriented roles and task-oriented roles independently and interactively (i.e., synergistically) predict ethical leadership. In the following sections, I discuss the theoretical implications, practical implications, strengths, limitations, and future research directions of the findings.

### Theoretical Implications

This paper makes three major theoretical contributions to advance ethical leadership literature. First, as previously discussed, extant research is largely focused on examining the consequences of ethical leadership compared to its antecedents. Despite some meaningful progress, the literature on the antecedents of ethical leadership still lacks the understanding of how ethical leaders balance (i.e., dynamic) between playing the role of relationship-oriented and task-oriented roles, which may have implications for ethical leadership in the organization (Rochford et al., 2017)—as such, putting too much attention toward one role while ignoring the other is potentially pernicious for ethical leadership. Results of the conducted studies indeed showed that both relationship-oriented and task-oriented roles synergistically predict ethical leadership. That is, relationship-oriented and task-oriented roles not independently but contingently optimize (i.e., synergistic interaction) the prediction of ethical leadership when both of them are at a high level. These findings provide empirical evidence and elaboration in favor of the arguments in theoretical pieces of Boyatzis et al. (2014) and Rochford et al. (2017) that our brain’s DMN and TPN networks consecutively constitute relationship-oriented and task-oriented leadership roles and a dynamic balance (i.e., synergistic interaction) between these two roles is required to be an ethical leader. Hence, the propositions drawn from opposing domain theory show a viable theoretical lens to expand the current understanding of the antecedents of ethical leadership. Indeed, such findings augment the organizational theorizing informed by the findings of neuroscientific research (Rochford et al., 2017).

Second, the findings of the present paper may offer a meaningful departure from the extant research in ethical leadership literature, which largely stresses cognitive/analytic reasoning as a basis for ethical leadership training and development (Brown & Treviño, 2006; Jordan et al., 2013; Wells & Schminke, 2001). In the present paper, I particularly expand this line of research by empirically demonstrating the importance of socioemotional reasoning (which is exerted through the demonstration of a relationship-oriented role) in contingent with analytic reasoning (which is exerted through the demonstration of a task-oriented role) as the antecedents of ethical leadership. Specifically, the findings show the presence of synergistic effects of relationship-oriented and task-oriented roles on employees’ perception of ethical leadership. These findings demonstrate that ethical leaders use neither of the leadership roles alone but both of them in a balanced way to be perceived as ethical leaders. Perhaps they have the ability to understand different cues around them, which helps them switch between socioemotional and analytical cognitive reasoning modes, correspondingly switching between their corresponding relationship-oriented and task-oriented roles as necessary (Boyatzis et al., 2014; French & Jack, 2015). In the same vein, these findings also shed initial light on scholars’ recent arguments that both moral reasoning and moral intuition (i.e., socioemotional reasoning) are required for developing an ethically-oriented leader (Egorov et al., 2019).

Third, the present paper’s findings can be deemed as a bridge to the research in psychology and neuroscience in the development of ethical leadership theorizing. In this paper, I tested and expanded the propositions developed based on a neurological theory at a psychological level, which permits interlevel synchronous investigation and provides explanatory robustness (de Jong, 2002). Hence, the present study findings demonstrate that the relationship between psychology and neuroscience concepts and data can be reconciled and complemented with each other as a way of a theory-building process in ethical leadership research.

### Practical Implications

This paper offers important insight to train leaders to be ethical leaders in the organization. Extant research in ethical leadership mainly suggests building leaders’ cognitive/analytic reasoning capacity to be ethical leaders (Jordan et al., 2013). The findings of the present research suggest that leaders can optimize their employees’ perception of ethical leadership if they uphold a high level of relationship-oriented and task-oriented roles, which correspond to their use of socioemotional and analytic reasoning. That is, they need to balance between their relationship-oriented and task-oriented roles by switching between those as necessary to be perceived as an ethical leader. Moreover, leaders need to effectively cycle back and forth between these two leadership roles to be ethical leaders, which can be trained by using different pedagogical techniques and experiential learning (see Boyatzis & Jack, 2018; Kolb, 2015; Rochford et al., 2017).

### Strengths, Limitations, and Future Research Directions

Like every study, despite having some strengths (e.g., pairing experimental and correlational designs), the current research is not free of a set of limitations that can be addressed in future studies. First, in the field study, I measured leaders’ relationship-oriented and task-oriented roles by employee ratings but not by the supervisors’ ratings. However, recent meta-analytic research has shown that leader-observer ratings were of similar magnitudes for relation- and task-oriented behaviors (Lee & Carpenter, 2018). Hence, I do not deem it problematic to interpret the study findings. Indeed, a future study can be carried out with the supervisor ratings of these roles. Second, I collected cross-sectional and single-source data for the field study, which may fall into the common method bias (Podsakoff et al., 2003). As I tested the interactive effect in this study, which is virtually impossible to create through common method variance (Siemsen et al., 2010), I did not expect common method variance to be a vital threat to the interpretation of the findings. Moreover, I found similar findings across the experimental and field studies designed with participants from different parts of the world (Western Europe and South Asia), which gave me the confidence to generalize my findings. Nevertheless, a future study can be carried out following multi-source data and a time-lagged design. Third, the measures of relationship-oriented and task-oriented leadership roles were previously used in experimental studies and not extensively validated for survey research, which is a potential limitation to acknowledge. However, I did CFA to provide evidence of the validity and distinctiveness of the measures (Conway & Lance, 2010). Indeed, I acknowledge that more research is needed to not only further validate (e.g., convergent validity) or refine the current field study measures but also carry out future research with other appropriate measures on this topic. Fourth, in this study I theorize based on the evidence informed by neurological measures but did not include any of those. Hence, caution is advised in interpreting the study findings. Indeed, it would be worthy to replicate the present study findings along with neurological measures to further increase the robustness of the same.

The present study findings offer some interesting avenues for future research. For example, organizational culture or climate or cultural congruence (i.e., as a moderator) may bind the leaders to be more susceptible toward one mode of reasoning over others, which may tend to make them toward excessive use of the corresponding leadership role over the other (Den Hartog & De Hoogh, 2024; Rochford et al., 2017).

### Conclusion

In conclusion, drawing on the propositions informed by opposing domain theory and related research, I examine leaders’ relational role maintenance behaviors with employees as the antecedents of ethical leadership. Specifically, across two studies, I show that leaders’ relationship-oriented roles and task-oriented roles synergistically predict employees’ perceptions of ethical leadership. I hope that the current investigation will act as a stepping stone to gain scholars’ interest in this promising direction.

## Data Availability

Data of the present research can be available upon reasonable request.
